# AhR Mediated Activation of Pro-Inflammatory Response of RAW 264.7 Cells Modulate the Epithelial-Mesenchymal Transition

**DOI:** 10.3390/toxics10110642

**Published:** 2022-10-27

**Authors:** Padhmavathi Selvam, Chih-Mei Cheng, Hans-Uwe Dahms, Vinoth Kumar Ponnusamy, Yu-Yo Sun

**Affiliations:** 1 Department of Medicinal and Applied Chemistry, Kaohsiung Medical University, Kaohsiung City 807, Taiwan; 2 Department of Biomedical Science and Environmental Biology, Kaohsiung Medical University, Kaohsiung City 807, Taiwan; 3 Department of Medical Research, Kaohsiung Medical University, Kaohsiung 804, Taiwan; 4 Research Center for Precision Environmental Medicine, Kaohsiung Medical University, Kaohsiung City 807, Taiwan; 5 Department of Marine Biotechnology and Resources, National Sun Yat-Sen University, Kaohsiung City 804, Taiwan; 6 Institute of BioPharmaceutical Sciences, National Sun Yat-Sen University, Kaohsiung City 804, Taiwan

**Keywords:** Aryl hydrocarbon receptor, macrophage, inflammatory cytokines, epithelial mesenchymal transition, MMP-9, Wnt/β-catenin

## Abstract

Pulmonary fibrosis, a chronic lung disease caused by progressive deterioration of lung tissue, is generated by several factors including genetic and environmental ones. In response to long-term exposure to environmental stimuli, aberrant tissue repair and epithelial cell-to- mesenchymal cell transition (EMT) trigger the subsequent progression of pulmonary fibrotic diseases. The Aryl hydrocarbon receptor (AhR) is a transcription factor that is activated by ligands providing lung dysfunction when activated by environmental toxins, such as polycyclic aromatic hydrocarbons. Our previous study demonstrated that AhR mediates α-SMA expression by directly binding to the α-SMA (fibroblast differentiation marker) promoter, suggesting the role of AhR in mediating fibrogenic progression. Here we follow the hypothesis that macrophage infiltrated microenvironments may trigger inflammation and subsequent fibrosis. We studied the expression of cytokines in RAW 264.7 cells by AhR activation through an ELISA assay. To investigate molecular events, migration, western blotting and zymography assays were carried out. We found that AhR agonists such as TCDD, IP and FICZ, promote the migration and induce inflammatory mediators such as TNF-α and G-CSF, MIP-1α, MIP-1β and MIP-2. These cytokines arbitrate EMT marker expression such as E-cadherin, fibronectin, and vimentin in pulmonary epithelial cells. Expression of proteins of MMPs in mouse macrophages was determined by zymography, showing the caseinolytic activity of MMP-1 and the gelatinolytic action of MMP-2 and MMP-9. Taken together, the present study showed that AhR activated macrophages create an inflammatory microenvironment which favours the fibrotic progression of pulmonary epithelial cells. Such production of inflammatory factors was accomplished by affecting the Wnt/β-catenin signalling pathway, thereby creating a microenvironment which enhances the epithelial-mesenchymal transition, leading to fibrosis of the lung.

## 1. Background

Pulmonary fibrosis, a chronic lung disorder [1], is indicated by the deposition of interstitial collagen and other extracellular matrix (ECM) components, as well as fibrotic modifications of lung parenchyma [2,3,4]. Fibrosis, a critical progression in general wound healing, is described as the abnormal extracellular matrix accretion and conformation, which substitutes for normal tissue [2]. It can be caused by connective tissue disease, environmental and occupational exposure such as silicosis and asbestosis, or drugs in patients with hypersensitivity pneumonitis [3]. In some cases, unusual ECM remodeling indicates the final phase of a chronic condition, leading to damage and failure of organs [5,6].

The activation of myofibroblasts and their differentiation is one of the crucial steps in the progression of fibrosis [7]. Inflammatory cells such as T-cells and macrophages are attracted to the site of inflammation when tissue damage subsequently leads to fibrosis [8]. The recruited cells generate a variety of pro-inflammatory cytokines, chemokines, and other angiogenic factors that facilitate the transition of epithelial cells to mesenchymal cells (EMT), activating effector cells which act as progenitors of cells such as myofibroblasts with mesenchymal characteristics, including high expression of α-SMA and the capability to produce the ECM [9]. The cellular process of epithelial cells transforming into cells with mesenchymal features by reducing epithelial cell markers; expression of E-cadherin while increasing α-SMA expression leading to EMT [2,10].

Macrophages are found in most tissues of the body and play important roles in both acute and chronic pulmonary pathologies such as cytotoxicity and fibrosis [11]. They belong to the more prevalent immune cells in most tissues, including the lungs, and are engaged in damaged tissue repair, wound healing, and metabolic activity regulation [7,12,13]. Macrophages release chemokines and cytokines such as IL-6, TNF-α, or matrix metalloproteins, which all promote inflammation. [14,15]. They can promote the expression of inflammatory factors such as TNF-α, which are primary mediators of an acute inflammatory response. Its overproduction is implicated in a number of pathological processes such as TCDD-related mortality, endotoxin hypersensitivity, and enhanced inflammatory response [16]. When researching for immunomodulatory and anti-inflammatory compounds, the RAW 264.7 cell line provides a well-known and reproducible inflammatory response, especially when challenged by inflammatory stimulants [17].

Due to industrialization, air pollution has become increasingly severe worldwide. [18]. At the cellular level, when a ligand attaches to the Aryl hydrocarbon receptor (AhR), the receptor is activated and governs the toxicity of environmental pollutants and polychlorinated dibenzo-*p*-dioxins such as the compound 2,3,7,8-tetrachlorodibenzo-p-dioxin (TCDD) [19]. Through AhR signalling, polycyclic aromatic hydrocarbon exposure increases the genesis and progression of lung cancer [20]. When AhR binds to TCDD, the translocation of AhR inside the nucleus occurs from the cytosol, causing modifications in AhR-targeted gene transcription with severe immunotoxicological effects [21,22]. The activation of AhR by TCDD was found to affect the expression of collagen and fibronectin, which are ECM proteins [23]. Matrix metalloproteinase (MMPs) expression, which is responsible for the destruction of ECM components, is expected to be a target of TCDD [24]. Matrix metalloproteinases (MMPs) as proteolytic enzymes contribute to the disintegration of the ECM [25]. MMPs are found to be calcium-dependent, zinc-containing endopeptidases that participate in cell migration, wound healing, and also tissue remodeling [26]. MMPs are closely linked to the promotion of cancer metastasis because they degrade the extracellular matrix [7]. 6-formylindolo [3,2-b]carbazole-FICZ is another endogenous AhR ligand, and is derived from tryptophan. It is a UV-induced photoproduct produced by the skin that has a high affinity to AhR. It is the utmost effective AhR ligand discovered to date.

FICZ is the most potent AhR ligand discovered to date. It binds to the human AhR with greater affinity than TCDD (TCDD K_D_ = 0.48 nM, FICZ K_D_ = 0.07 nM) [19,27]. In this study, we have compared the effects of TCDD and FICZ to the specificity of IP as an AhR agonist, a potential component of ambient polycyclic aromatic hydrocarbon present in the southern Taiwan environment [28].

In normal epithelial cells, β-catenin is a component of basic adherens junctions and is cardinal to bind to the cadherin cytoplasmic tails of the cytoskeleton, providing cell to cell adhesion [29]. The Wnt/β-catenin signalling system is evolutionarily conserved, performing a vital role in the development of embryonic regulation, cell proliferation, motility, wound healing, tissue homeostasis, and stem cell self-renewal [30]. In the developing lung, Wnt signalling is frequently reactivated in mature lungs throughout injury restoration and tissue repair [31,32].

At certain conditions, β-catenin piles up in the cytoplasm owing to Wnt pathway activation or, any aberration in the β-catenin degrading machinery, β-catenin is translocated to the nucleus [33]. In the nucleus, β-catenin binds itself to the members of the TCF-T-cell factor/LEF-lymphocyte enhancer factor transcription factor family acting as a transcriptional coactivator, where it helps in promoting the transcription of the EMT-regulating genes, vimentin and fibronectin. As a result, the accumulation of β-catenin inside the nucleus has been linked to the migration of epithelial cells and EMT in pathological conditions such as tumor progression [34]. Chronic AhR activation alters cardiomyocyte function and promotes fibrosis via the disruption of the Wnt pathway [35].

Recent research found that the Wnt/β-catenin pathway is important to initiate Wnt target gene transcription, including fibrosis-associated gene expression, such as fibronectin [34,36]. The β-catenin-signalling pathway could be a new therapeutic target for fibrotic illnesses [37,38]. AhR can promote the β-catenin-mediated epithelial-mesenchymal transition, which promotes the initiation and progression of lung fibrosis, by regulating relevant biochemical mechanisms such as phosphorylation via downstream components [39,40,41].

An open question is whether connections in between AhR and the Wnt/β-catenin signalling pathways contribute to the toxicity mediated by AhR agonists in the macrophage associated microenvironment. We tested the hypothesis that the treatment of macrophages with an AhR agonists results in the release of inflammatory cytokines, thereby creating a microenvironment which leads to epithelial cell-to-mesenchymal cell transition. This follows the Wnt/β-catenin signalling path, leading to fibrosis. We focus here on the significance of AhR signalling in macrophage activation and migration, ECM breakdown, and also on the generation of proinflammatory cytokines, which are hypothesized to have a vital role in fibrosis progression.

## 2. Materials and Methods

### 2.1. Chemicals, Reagents and Antibodies

Ham’s F12 medium, Dulbecco’s Modified Eagle’s Medium (DMEM) (high glucose), fetal-bovine serum (FBS; heat inactivated), L-glutamine, HEPES, insulin-transferrin-selenite (ITS), estrogen, hydrocortisone, penicillin-streptomycin solution, and sodium pyruvate solution was obtained from Gibco (Gaithersburg, MD, USA). The AhR agonists, TCDD and CH223191, were obtained from Sigma-Aldrich (St. Louis, MO, USA). IP was purchased from Supelco^®^ and FICZ was obtained from Enzo Life Sciences, Inc., Farmingdale, NY, USA. Gelatin from porcine skin and casein was purchased from Sigma (Sigma-Aldrich, St. Louis, MO, USA). For western blotting, the following antibodies were utilized: anti-E-cadherin (#3195), β-catenin (#8480), anti-rabbit IgG–HRP (#7074) and anti-mouse IgG–HRP (#7076). All of these compounds were purchased from Cell Signalling (Cell Signalling Technology, Danvers, MA, USA), while anti-Vimentin (GTX00619) and anti-GAPDH (GTX100118) were purchased from Gene Tex (GeneTex, Irvine, CA, USA). Anti-Fibronectin (AB1954-25UL) was obtained from Merck (Darmstadt, Germany).

### 2.2. Cell Line Treatment

Mouse macrophage cell lines (RAW 264.7) were collected from the Bioresource Collection and Research Centre (Hsinchu, Taiwan). Mouse lung epithelial cell lines MLE-12 were purchased from ATCC (CRL-2110). RAW 264.7 cells were sub-cultured routinely in DMEM along with FBS—10%- and 2-mM sodium pyruvate and L-glutamine. MLE-12 was sub-cultured in DMEM/F12 50:50. Three AhR agonists TCDD (Sigma-Aldrich, St. Louis, MO, USA), IP (Supelco^®^) and FICZ (Enzo Life Sciences, Inc., Farmingdale, NY, USA) were used in our study. DMSO (Sigma) was applied to dissolve all compounds and used as a DMSO control (0.1%).

### 2.3. Chemokine-Cytokine Screening

To investigate the immunological response elicited by the AhR agonist-TCDD in RAW 264.7 cells, 1 × 10^5^ cells/mL cells were seeded at ~ 90% confluence on 60 mm plates and cultured for 24 h at the same conditions as mentioned above [13]. The cytokine/chemokine expression profiles in RAW 264.7 cells induced by TCDD were analyzed and assessed to control cells treated with DMSO (Kuo, Yang et al. 2021). According to the manufacturer’s instructions, the Proteome ProfilerTM Mouse Cytokine Array Panel A (Catalog Number ARY006) was used to analyse the retrieved culture supernatant (R&D Systems, Minneapolis, MN, USA). The array image was quantified using Image J software.

### 2.4. Enzyme-Linked Immunosorbent Assay

RAW 264.7 cells (1 × 10^5^ cells/mL) were cultivated to confluence in 96-well plates [12]. The cells were treated with control and AhR agonists TCDD, IP, FICZ for 24 h at 37 °C. For treatments with AhR antagonists, RAW 264.7 cells were pre-treated with CH223191 for 2 h before AhR agonist treatment. After treatment, the cell supernatant was collected by centrifugation at 1300 rpm for 3 min (Eppendorf centrifuge-5810) and used for the measurement of MIP-2 (Catalogue number: DY452-05) and TNF-α (Catalogue number MTA00B), G-CSF (Catalogue number DY414-05), MIP-1α (Catalogue number: DY450-05) and MIP-1β (Catalogue number: DY451-05). Based on the manufacturer’s protocol (R&D Systems), the absorbance was determined by a Rayto RT-6900 microplate reader (Shenzhen, China).

### 2.5. Cell—Migration Assay

For cell migration studies, a modified 24-well Boyden chamber was used. The transwell upper chamber, with a pore size of 8.0 μm membrane (BD Discovery Labware, Billerica, MA, USA), was placed into the plates. The lower chamber contained SF media with AhR agonists. The upper part of the chamber contained 300 μL of serum-free medium with RAW 264.7 mouse macrophage cells (2 × 10^5^ cells/mL) or MLE-12 cells (5 × 10^4^ cells/mL), respectively, and an AhR agonist was added to the lower part of the chamber and incubated at 37 °C for 24 h. The RAW 264.7 treated conditioned medium was obtained as follows: 2 × 10^5^ cells/well of RAW 264.7 cells were seeded and treated with an AhR agonist, and this medium was collected and used in the bottom chamber, whereas (5 × 10^4^ cells/mL of MLE-12 cells were seeded in the upper part of the chamber [42]. The cells migrating across the filter were detected using crystal violet staining (see Fachin et al. 2022).

### 2.6. Crystal Violet Staining

The media were removed 24 h after the addition of cells to the upper part of the transwell chamber, and PBS was used to wash the cells. Methanol (500 µL) was applied for 15 min to fix the samples. The upper chamber of the transwell was rinsed thrice using PBS, and 450 μL of 1% crystal violet (Sigma-Aldrich) suspended in 20% methanol was applied to stain the cells of the upper chamber’s bottom. Cell fixation was done for 30 min with 20% methanol. Excess stain was removed by washing with PBS [43]. The cells were photographed and counted in three to five fields of the chamber at random.

### 2.7. Zymography

The gelatinolytic and caseinolytic activities of macrophage cultures were determined by performing zymography following a similar method as previously described [12]. 1 × 10^5^ cells/mL of RAW 264.7 macrophage cells were grown in a 60 mm dish to confluence. The cells were treated with control and AhR agonists TCDD, IP, FICZ for 24 h at 37 °C. Amicon Ultra centrifugal filter units Ultra-15 of MWCO 10 kDa were used to collect and concentrate the supernatant. For zymography, 16 µL of each concentrated medium were scaled down to represent equal amounts of cellular protein (15 mg). The sample was treated with a non-denatured sample buffer. For MMP-2 and MMP-9 analysis, a 0.1% gelatin incorporated 7.5% polyacrylamide gel was used. For MMP-1 analysis, a 1% casein incorporated 7.5% polyacrylamide gel was used. For SDS removal, the PAGE gels were washed twice at room temperature for 15 min, using 2.5% Triton X-100. The gel was then immersed in a buffer for development [50 mM Tris-HCl (pH 7.5), 200 mM NaCl, 5 mM CaCl_2_ and 0.25% Brij-35] and 37 °C overnight incubation to permit the substrate’s digestion of the proteinases. Following this, the gel was stained using 0.5% Coomassie brilliant blue R-250 (40% methanol and 10% acetic acid) for 30 min. A destaining was performed with 40% methanol and 10% acetic acid. The appearance of bands of clear lysis against the dark background indicated proteolytic activity. The bands were quantified using Image J software.

### 2.8. Western Blotting

In 60 mm plates, MLE-12 cells amounting 5 × 10^5^ cells/well were seeded in a culture medium at a confluence of 90% and treated with AhR agonists and antagonists as specified above and using a PBS washing process. Afterwards, a RIPA cell lysis buffer was used for cell lysis. A cytoplasmic and nuclear extraction kit was used for cytosolic and nuclear protein extraction (EMD Merck Millipore, Darmstadt, Germany). The concentration of protein was determined using a BCA protein assay kit (Thermo Fisher, Shanghai, PR China). Using 10–12 percent SDS-PAGE, equivalent amounts of protein were separated. After this, the material was transferred to a nitrocellulose membrane (Amersham Biosciences). TBST-Tris buffered saline-tween20 (20 mM Tris-HCl at pH 7.4, 150 mM NaCl, 0.1% Tween-20), comprising 5% non-fat milk, was used to block the membrane. BSA/TBST (1%) was used as a diluent for primary antibody incubation. It was then incubated in 5% non-fat milk HRP-conjugated secondary antibodies. The image was developed through chemoluminescence [24] and was quantified using Image J software.

### 2.9. Statistics

The values are based on the results of at least three separate studies in each condition ± SEM. One-way analysis of variance (one way ANOVA) was used to analyze the data. Differences were deemed as significant when: * *p* < 0.05, ** *p* < 0.01, *** *p* < 0.001, and **** *p* < 0.0001.

## 3. Results

### 3.1. TCDD Promotes Expression of Cytokine and Chemokine in Macrophage RAW 264.7 Cells

**Figure 1:**
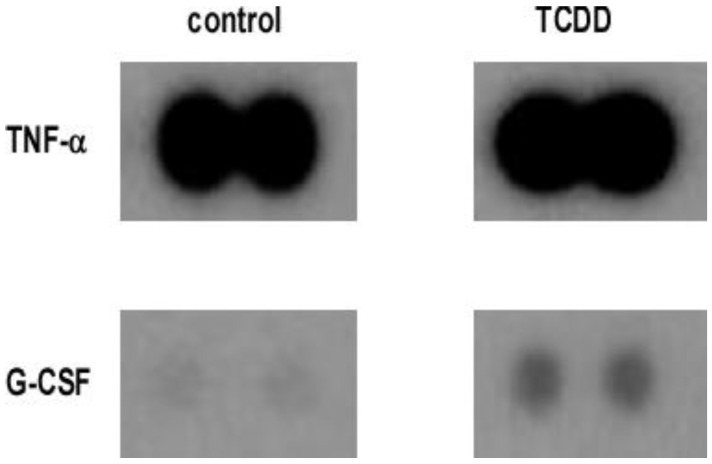
Chemokine-Cytokine screening. Activation of AhR in macrophage induced inflammatory markers and fibrosis-associated genes. RAW 264.7 cells were nursed in serum-free DMEM media with TCDD (10^−8^ M) for 24 h. The cytokine/chemokine expression profiles in RAW 264.7 cells induced by TCDD were analyzed and compared to control cells that were treated with DMSO.

The cell signals and the inflammatory response are generally mediated by a number of cytokines [20]. Previous reports indicated that the immune response elicited by TCDD was moderated through the production of the cytokines upon AhR activation, which plays a crucial role in the process of inflammation [16,22]. To study the effects of TCDD on AhR activation, we tested whether the expression of the released pro-inflammatory cytokines was related to TCDD, thereby leading to the activation of AhR through the release of cytokines in the macrophage. A cytokine array assay (Supplementary Figure S1) was used to determine AhR activation and the consequent release of several pro-inflammatory cytokines. TCDD was used to treat RAW 264.7 cells. As described above, multiple cytokine levels were determined from a single sample. The results showed an increased level of cytokines, the majority being TNF-α and G-CSF (Figure 1). Additionally, increased levels of cytokines included macrophage inflammatory proteins such as MIP-1, MIP-1, and MIP-2 (Supplementary Figure S2). Pro-inflammatory cytokines were secreted by RAW264.7 cells upon activation of AhR by the treatment with TCDD, further suggesting the role of AhR in modulating an inflammatory response.

### 3.2. AhR Activation by Agonists Promotes the Production of Cytokines

**Figure 2:**
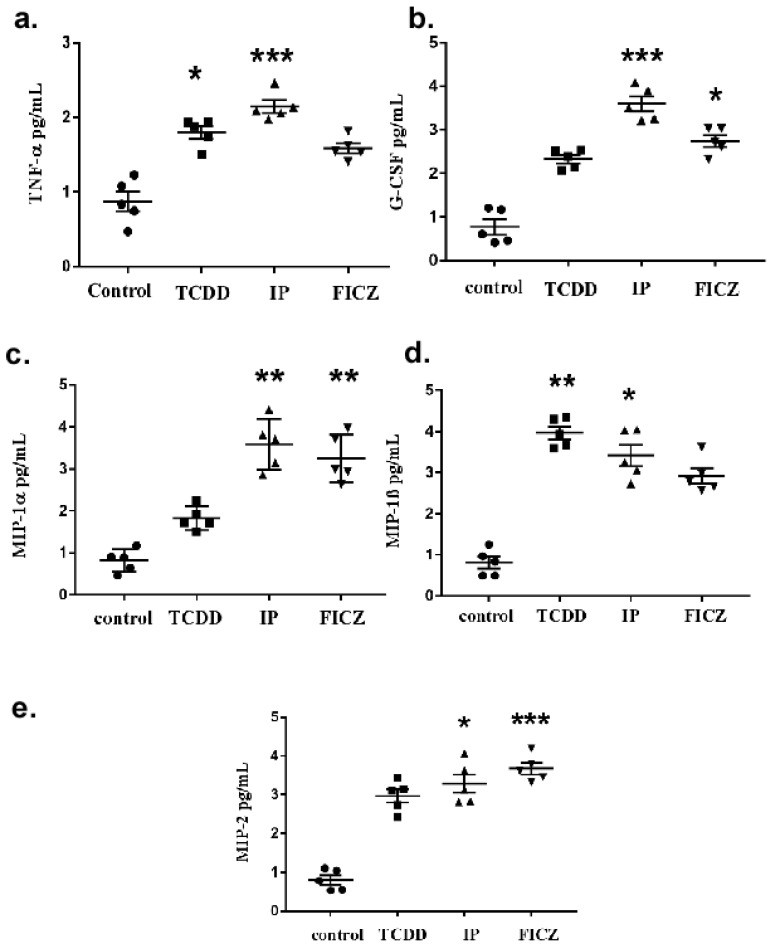
Activation of AhR in macrophages induces inflammatory markers and fibrosis associated cytokines. In a serum-free DMEM medium, RAW 264.7 cells were cultured for 24 h with TCDD (10^−8^ M), IP (10^−7^ M), and FICZ (10^−7^ M). An increase in cytokine levels is shown as folds of increase compared to the control culture. (**a**) TNF-α expression, (**b**) G-CSF expression, (**c**) MIP-1α expression (**d**) MIP-1β expression, (**e**) MIP-2expression Three independent experimental means ± SEM are represented in the graph. Unstimulated comparisons to control cultures were depicted by asterisks, * *p* < 0.05, ** *p* < 0.01 and *** *p* < 0.001 by one-way ANOVA analysis.

The observation that TCDD induced the expression of cytokines and chemokines upon activation of AhR let us speculate whether cytokines would subsequently be induced by other AhR ligands such as IP and FICZ. To prove this, the effects of AhR agonists were evaluated using ELISA assays, which quantified the produced cytokines individually. The effects of AhR agonists on the secretion of cytokines G-CSF, TNF-α, MIP-1α, MIP-1, MIP-2 in RAW 264.7 cells were analyzed by ELISA assay. As illustrated in Figure 2a, all three AhR agonists significantly induced the expression of TNF-α. A comparison showed that IP increased its level by 0.7-fold and was the most upregulated agonist. As seen in Figure 2b, IP increased the production of the G-CSF cytokine by 0.7-1-fold compared to TCDD and FICZ.

In the case of the macrophage inflammatory proteins, all three AhR agonists were found to be significantly increased, but a comparison among them provided varied results. As shown in Figure 2c, both IP and FICZ-treated cells secreted equal levels of MIP-1α cytokine. However, as shown in Figure 2d, TCDD treated cells secreted slightly increased levels of MIP-1β cytokine. Also, in Figure 2e, FICZ treated cells increasingly secreted MIP-2 cytokine compared to TCDD and IP.

### 3.3. RAW 264.7 Cell-Conditioned Medium Induces Mouse Lung Epithelial Cell Migration

**Figure 3:**
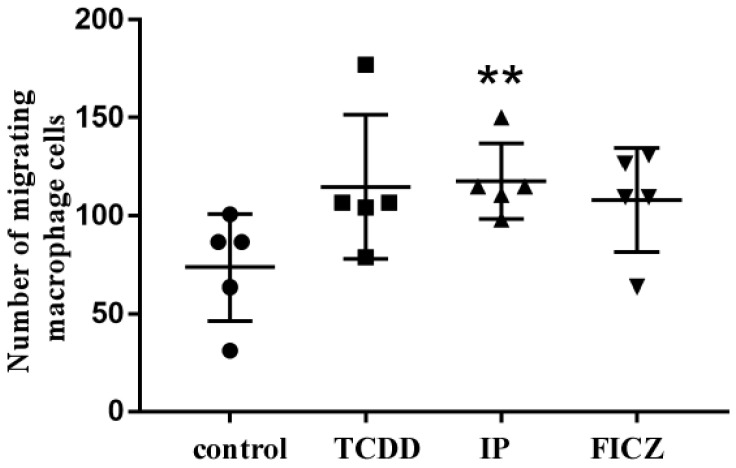
Migration of macrophage cells. In serum-free DMEM media with TCDD (10^−8^ M), IP (10^−7^ M) and FICZ (10^−7^ M), RAW 264.7 cells were cultured for 24 h, and images were taken. Crystal violet-stained cells take on a purple hue. The quantification of cells was performed as described above. The number of cells that migrated were totaled and presented as folds of increase compared to the control cells. Five independent experimental means ± SEM are represented in the graph. Unstimulated comparisons to control cultures were depicted by asterisks, such as. ** *p* < 0.01 by one-way ANOVA analysis.

**Figure 4:**
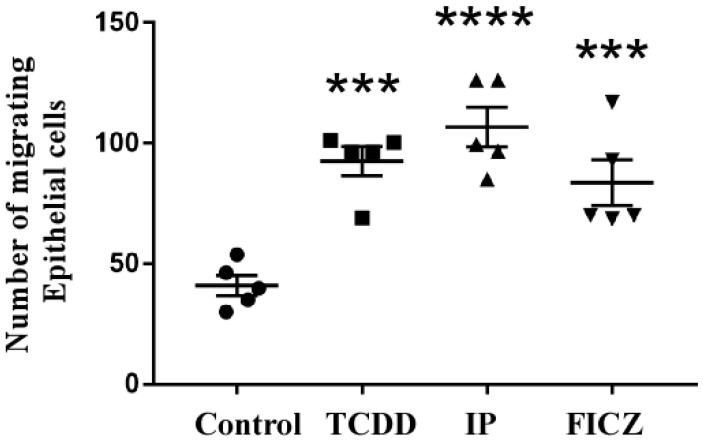
Migration of epithelial cells. In serum-free DMEM/F12 medium that contained TCDD (10^−8^ M), IP (10^−7^ M), FICZ (10^−7^ M^)^, MLE-12 cells were cultured for 24 h. Images were taken after 24 h. Crystal violet-stained cells took on a purple hue. The quantification of cells was performed as described above. The number of migrating cells was totaled and presented as folds of increase compared to control cells. Five independent experimental means ± SEM are represented in the graph. Unstimulated comparisons to control cultures were depicted by asterisks, such as *** *p* < 0.001 and **** *p* < 0.0001 by one-way ANOVA analysis.

**Figure 5:**
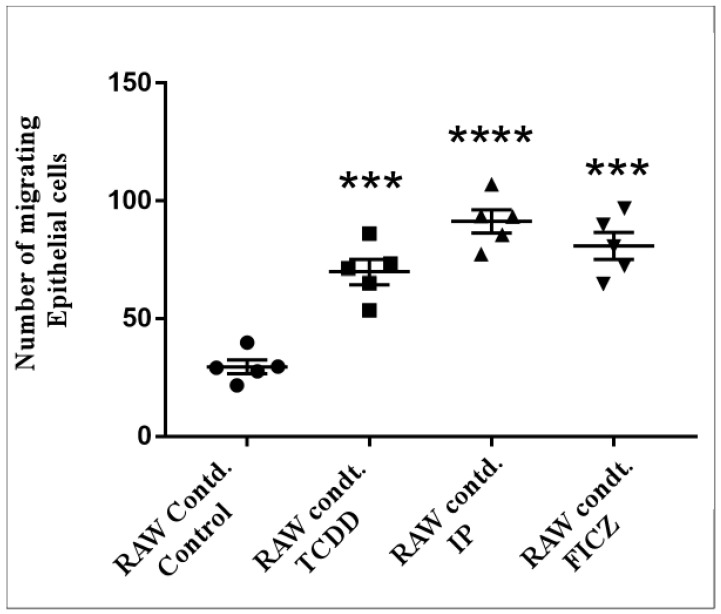
Effect of conditioned medium. In serum-free DMEM media with TCDD (10^−8^ M), IP (10^−7^ M), FICZ (10^−7^ M) for 24 h, RAW 264.7 cells were maintained. MLE-12 cells were seeded for 10–12 h before treatment for 24 h with RAW 264.7 conditioned medium. Images were taken after 24 h. Crystal violet-stained cells took on a purple hue. The quantification of cells was performed as illustrated previously. The number of cells that migrated were totaled and presented as folds of increase compared to the control cells. Five independent experimental means ± SEM were represented in the graph. Unstimulated comparisons to control cultures were depicted by asterisks, such as *** *p* < 0.001 and **** *p* < 0.0001 by one-way ANOVA analysis.

Increased motility is a characteristic of epithelial cells that experienced EMT (epithelial mesenchymal transition). A general marker for EMT differentiation is cell migration [14]. To determine the influence of AhR ligands in mouse macrophages (Supplementary Figure S2) and mouse pulmonary epithelial cells (Supplementary Figure S3) individually, a Boyden chamber-oriented cell migration assay was carried out. As shown in Figure 3, upon treatment with AhR agonists, the migration of macrophage cells was observed, where IP was found to be more effective than TCDD and FICZ. These results were similar in the case of epithelial cell migration (Figure 4). IP was found to be most effective in both cases. To test our hypothesis that the release of cytokines through the activation of AhR by macrophages had an effect on epithelial cells, we treated epithelial cells with a RAW 264.7 cell-conditioned medium. Macrophage cells were treated for 24 h in serum-free culture medium containing TCDD (10^−8^ M), IP (10^−7^ M), and FICZ (10^−7^ M). MLE-12 cells were seeded for 10–12 h and were treated with RAW 264.7 conditioned medium for 24 h (Supplementary Figure S4). As shown in Figure 5, IP was found to be upregulated significantly higher compared to TCDD and IP. The above results demonstrate that epithelial cell migration is influenced by pro-inflammatory cytokines that induced a microenvironment created by macrophages upon activation by AhR.

### 3.4. Activation of AhR Induces MMP Expression in Macrophage RAW264.7 Cells

**Figure 6:**
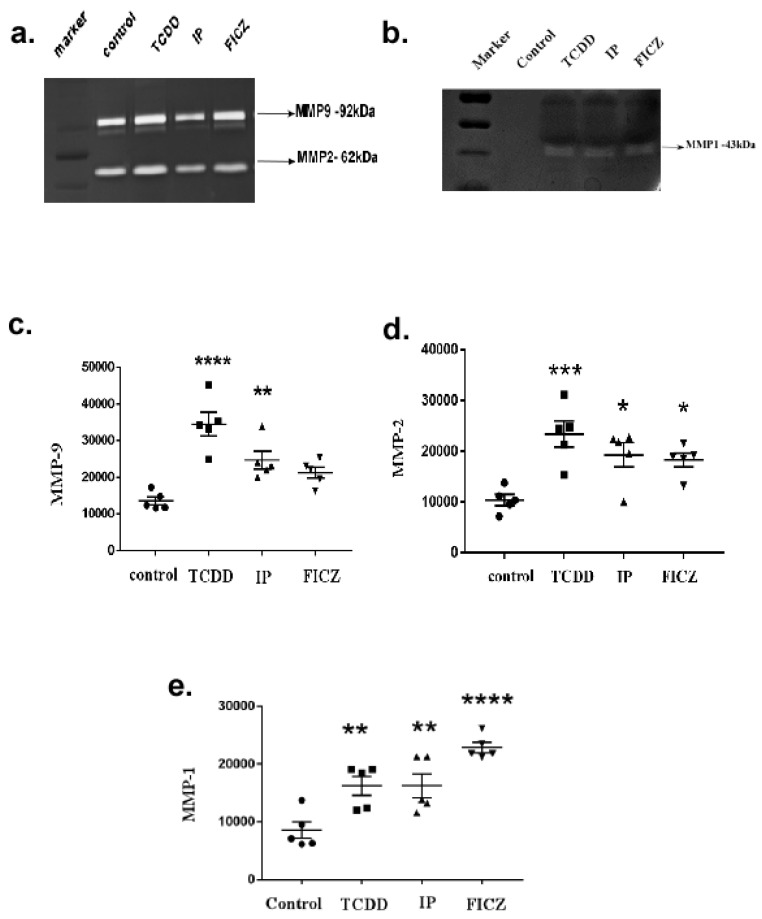
Activation of AhR induced MMP expression in RAW264.7 cells. In serum-free DMEM media with TCDD (10^−8^ M), IP (10^−7^ M), and FICZ (10^−7^ M) for 24 h, RAW 264.7 cells were incubated. The cell supernatant was collected and subjected to SDS-PAGE (containing 0.1% gelatin or 1% casein) electrophoresis. (**a**) Representative image of gelatin zymogram. (**b**) Representative image of casein zymogram. Induction of RAW 264.7 cells with AhR agonists produced (**c**) MMP-9, (**d**) MMP-2, (**e**) MMP-1 expressions. The levels of secreted MMPs are shown as folds of increase compared to the control. Means of five independent experiments +/-SEM were represented in the graph. Unstimulated comparisons to control cultures were depicted by asterisks, such as * *p* < 0.05, ** *p* < 0.01, *** *p* < 0.001 and **** *p* < 0.0001 by one-way ANOVA analysis.

MMP activity is central to ECM remodeling injury [8]. The first linkage between AhR and the extracellular matrix (ECM) was uncovered in studies with TCDD exposure [2]. The initiation of the AhR pathway with TCDD was shown in previous animal models to trigger MMP overexpression; meanwhile, MMP downregulation is caused by the loss of AhR, which consequently resulted in abnormal matrix metabolism and the migration of epithelial cells [44]. Collagenases break the natural helix of fibrillar collagens after fibrotic liver damage, producing gelatin that MMPs can destroy, namely MMP-2 and MMP-9 [25,45,46,47] (Supplementary Figures S5 and S6). To determine whether macrophage cells, upon treatment with AhR agonists, results in MMP secretion, we checked the expression of MMPs in the culture medium by performing a substrate gel zymography assay (Figure 6a). The substrate gelatin gel zymography revealed two major clear bands of proteolytic activity at 72–75 kDa, which corresponds to MMP-2 (Figure 6d). At 94–100 kDa it corresponds to MMP-9 (Figure 6c). No significant difference in quantities among the control and experimental group was observed. However, TCDD treatment was found to substantially increase the secretion of MMP-2.

An additional clear band of proteolytic activity at 43 kDa was identified as MMP-1 in a casein-gel zymography (Figure 6b). However, among the treatment groups only MMP-1 was significantly upregulated by FICZ (Figure 6e).

### 3.5. AhR Activation Induces EMT Marker Expression in Mouse Lung Epithelial Cells

**Figure 7:**
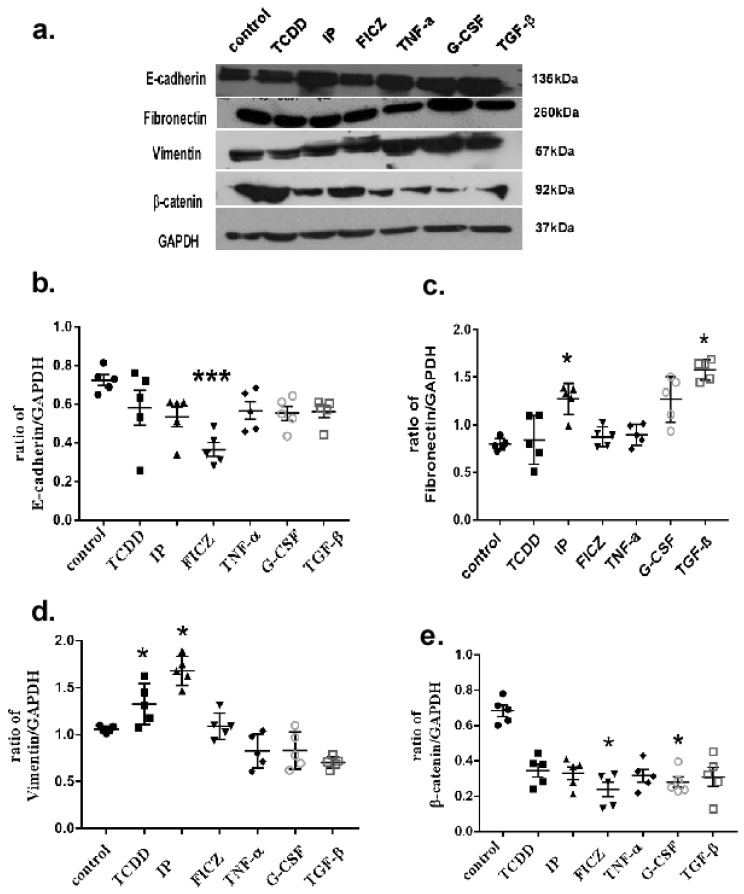
Activation of AhR induced epithelial EMT marker expression in MLE-12 cells. MLE-12 cells were exposed for another 24 h in serum-free DMEM/F12 media with TCDD (10^−8^ M), IP (10^−7^ M), FICZ (10^−7^ M), TNF-α (10 ng/mL), TGF-β (10^−5^ M) and G-CSF (10 ng/mL). (**a**) Representative image of western blot. (**b**) e-cadherin expression, (**c**) fibronectin expression, (**d**) vimentin expression, (**e**) β-catenin expression. Five independent experimental means ± SEM are represented in the graph. Unstimulated comparisons to control cultures were depicted by asterisks, such as * *p* < 0.05 and *** *p* < 0.001 by one-way ANOVA analysis.

Epithelial-to-mesenchymal transition (EMT) is defined as the process where cells lack their cell-to-cell adhesion and polarity and turn into cells with mesenchymal properties [48]. They then decrease their epithelial characteristics, likely having low E-cadherin expression while increasing the expression of α-SMA. To further examine the association and the impact between AhR agonists and cytokines released by macrophage cells on epithelial cells, a western blotting assay was performed. Epithelial cells were treated with AhR agonists and cytokines from the previous results, such as TNF-α and G-CSF (Supplementary Figures S7–S11). Figure 7a represents the blot image. In this study, in case of E-cadherin (Figure 7b), an epithelial cell marker was downregulated upon treating mouse epithelial cells with AhR agonists. IP, FICZ along with the cytokine G-CSF was found to be the most significant agonist in the downregulation of E-cadherin. When observing the results of fibronectin (Figure 7c) and vimentin (Figure 7d), mesenchymal markers and IP was found to upregulate both proteins along with the G-CSF.

β-catenin is an important factor for cell-to cell adhesion, where it operates as a potent linkage amid E-cadherin and the cytoplasmic actin cytoskeleton [36]. It is the central part of the Wnt/β-catenin signal pathway, and under aberrant conditions, it functions as a coactivator of transcription, thereby promoting transcription of genes to cause physiological changes in cells, such as EMT [32,36]. The reduction of E-cadherin expression causes the disruption of the cadherin and the catenin complex, thereby resulting in the increased accumulation of β-catenin in the nucleus. In this study, upon AhR agonist treatment, in the cytosolic fraction of protein, IP was found to be a more effective agonist along with the cytokine TNF-α, as it resulted in β-catenin accumulation in the cytoplasm. Taken together, these results demonstrated that the epithelial cell to mesenchymal transition ensued as a result of AhR activation by the AhR agonist treatment via Wnt/β-catenin pathway activation.

## 4. Discussion

Polycyclic aromatic hydrocarbon (PAH) exposure is known to promote lung cancer development via AhR signalling [9]. The function of AhR in mediating the effects of environmental stressors was discovered first by Cui et al. [1]. However, with the intervention of several other ligands which are endogenous and a better understanding of AhR functioning in recent years, AhR was shown to display a key role in establishing standard physiological activities [49,50]. It is generally assumed that pulmonary fibrosis is caused by the repetitive aberrant repair of pulmonary epithelial tissues [5]. Compared to the overall number of macrophage studies, only a few focus on the interaction between macrophages and epithelial cells. As a result, we investigated the potential involvement of cytokines generated by macrophage cells following AhR activation by AhR agonist treatment, which could lead to epithelial-mesenchymal transition.

Our data showed that cytokine production by macrophages through AhR-activation, in part, induces pulmonary epithelial cell migration and differentiation. The present study pursued how AhR agonist treatment affected the expression of molecules involved in ECM metabolism during lung fibrosis. Consistent with the literature [12,16], our results showed that the CYP1A1 and CYP1B1 (Supplementary Table S1, Supplementary Figure S12) cytokines were induced upon treatment with TCDD. According to our findings, cytokines such as MIP-1α, MIP-1β, and MIP-2 and G-CSF were also elevated in addition to TNF-α. Normal inflammation protects the body, but abnormal inflammation causes a variety of inflammatory diseases [5]. The main contributor to pulmonary fibrosis in the lung tissues is an abnormal defensive mechanism that involves of inflammation, healing, and structural remodeling. Furthermore, lymphocytes, macrophages, neutrophils, and eosinophils are all activated at early-stage acute lung inflammation, which then promotes the autoimmune response by secreting cytokines and inflammatory mediators [11]. Cytokines are tiny proteins (5–20 kDa) that play a significant role in immune system communication, allowing host tissues and immune cells to exchange information and interact as peptides in cell signalling. Chemokines are a type of cytokine that cause immunological cells to migrate to the place of an injury, known as the chemotaxis of immune cells [51]. The combined effect of cytokines and chemokines, render them as essential factors in the process of tissue repair following infection or injury. When macrophages trace an infection, TNF-α is released, which attracts other components of the immune system and assists in the immune response. TNF-α binds to a range of immune system components, including Interleukin-1 and Interleukin-6, and triggers the production of chemokines like MIP-1α, MIP-1β, and MIP-2, which are small cytokines released by monocytes and macrophages that belong to the C-X-C motif chemokine family [5,14,45].

In the present study, all three AhR agonists, TCDD, IP and FICZ, induced the release of the cytokines TNF-α, MIP-1α, MIP-1β, and MIP-2. IP was found to markedly enhance the level of cytokines when compared to other agonists. Our hypothesis is supported by these results. Accumulating evidence revealed that macrophages enhance the migration and cytokine release and therefore, lead to pulmonary fibrosis. Recent studies indicated that macrophages boost the advancement of pulmonary fibrosis [30,38]. Since the migration of epithelial cells is the first step in the process of pulmonary fibrosis, we treated both macrophage and epithelial cells with AhR agonists. Here, IP showed the strongest effect on both macrophage (Figure 3) and epithelial cell migration (Figure 4). When a macrophage conditioned medium was used in the treatment of epithelial cells, migration was induced by all three agonists; however, IP was found to have the strongest effect (Figure 5).

A multitude of cytokines and growth hormones, such as IL-1 and TNF-α, affect MMP transcription [51]. A recent study revealed that AhR signalling regulates MMPs, which play a major role in ECM deposition and the metabolism of its components [51]. The results of the present study indicate a substantial increase of MMP-2, MMP-9 and MMP-1 by all three AhR agonists. In agreement with the above results, we found that TCDD significantly induced MMPs compared to FICZ and IP. In particular, MMP-1 was found to be significantly more highly expressed by FICZ.

EMT is required for embryogenesis, chronic inflammation, and tumor progression, as well as wound healing. Abnormal EMT can lead to fibrosis. The cells changed during this phase, affecting their physiological functions, migration and adhesion in concert with basal and neighbouring cells [44]. The enhanced expression of EMT markers indicate epithelial cells that have undergone EMT. A series of molecular mechanism events have been discovered for epithelial cell-mesenchymal cell transition, including E-cadherin suppression, fibronectin and vimentin overexpression, and the interruption of adherens junctions [2,14]. We demonstrated with our results that AhR agonists induce EMT. Our results also show that fibronectin and vimentin (mesenchymal markers) were upregulated. Compared to TCDD and FICZ, IP was found to induce mesenchymal markers to a larger extent, whereas, FICZ significantly downregulated E-cadherin, and IP was found to upregulate β-catenin compared to TCDD and FICZ. A direct treatment of TNF-α did not affect the E-cadherin down-regulation as compared to the treatments of IP, FICZ and G-CSF, suggesting that a combined effect of cytokines and chemokines are required in downregulating E-cadherin. 

Cadherins are receptors for transmembrane adhesion that facilitate homophilic associations by establishing adherent bindings in between either one or more domains of the immunoglobulin in their extracellular area. They also link to actin microfilaments in an indirect manner in the cytoplasm through α and β-catenin. They encourage the creation of adherens junctions and the formation of permanent cell-cell interactions [44]. The characteristic feature of the Wnt/β-catenin signalling activation in epithelial cells is ß-catenin accumulation in the cytoplasm. Wnt signalling is triggered by the development of a trimeric ligation complex including Wnt/Fzd/co-receptors. There are three types of Wnt signalling pathways which could be initiated based on the complex’s makeup [52]. They include the canonical Wnt/β-catenin route, which has a β-catenin dependent pathway; a non-canonical route that is not dependent on β-catenin; and the Wnt-Ca^+2^ route, which is where protein kinase C (PKC) is initiated. The activation of the canonical pathway, namely β-catenin/TCF-dependent pathway is heavily reliant on β-catenin [29,52]. The signalling cascade and other ligands of Wnt such as prostaglandin and E-cadherin, were discovered to operate as β-catenin extracellular regulators [53]. Results obtained from our western blot assay indicated the accumulation of β-catenin in the cytoplasm upon treatment with the AhR agonist IP. However, when comparing the control and experimental groups, higher β-catenin was observed in the control group, which suggests that the activation of AhR may promote the translocation of β-catenin into the nucleus initiating the Wnt/β-catenin signalling pathway. Yet, we did not observe an enhanced β-catenin nuclear expression (data not shown). A similar study also found that TCDD had no effect on β-catenin nuclear accumulation, and using a western blot assay, they were unable to observe the expression of other proteins such as snail1, snail2, twist, and ZEB1 [44]. However, Shiizaki et al. reported that through the substitution of amino acids or by treatment with LiCl, translocation of β-catenin to the nucleus was possible, enhancing AhR-mediated CYP1A1 induction [34]. According to their findings, there has to be a positive feedback loop between β-catenin signalling and the functioning of AhR [34].

A few studies also suggest that unusual Wnt-β-catenin signalling pathway activation happened to be critical for regulating the pulmonary fibrogenesis process and to cause fibroblasts and mesenchymal stem cells of lungs to transform into myofibroblasts initiating lung fibrosis by increasing collagen fiber secretion and ECM deposition. It was discovered that in bronchiolar lesions, β-catenin nuclearizes abnormally increased EMT and promoted pulmonary fibrogenesis in injured lungs [39,48,54,55].

Our study discovered that treatment with AhR agonists stimulated macrophage cells and induced cytokine production and ECM degradation. We confirmed that pro-inflammatory cytokines released by the macrophage cell initiate the EMT process and macrophages were important for the progression of pulmonary fibrosis relying on AhR agonists. The present study used a cytokine array to discover cytokines that were differently expressed after AhR agonist treatment in macrophage cells to investigate mechanisms of EMT and pulmonary fibrotic progression. Remarkable upregulations of TNF-α, G-CSF, MIP-1α, MIP-1β and MIP-2 (Figure 2) were demonstrated. MMP-2, MMP-9, and MMP-1, all of which are involved in ECM breakdown, were also enhanced. Our results indicate that the Wnt/β-catenin pathway was highly stimulated during the advancement of the EMT process.

The present study signifies that β-catenin in Wnt/β-catenin signalling is responsible for the pathogenesis of pulmonary fibrosis and fibrotic disease progression. Collectively, our study explored EMT progression induced by pro-inflammatory cytokines released by macrophages upon AhR activation and the role of Wnt/β-catenin in pulmonary fibrosis pathogenesis. Whether there is a cross-talk between MMP signalling and the machineries of the Wnt-β-catenin pathway along the AhR axis remains to be solved.

## 5. Conclusions

The activation of the Aryl hydrocarbon receptor induces pro-inflammatory cytokines, which along with potential AhR agonists are responsible for creating a microenvironment that promotes the migration of macrophages and mediates the expression of EMT biomarkers. In particular, we found that macrophages promote EMT through activating Wnt/β-catenin signalling. In order to identify the mechanisms or the pathways involved in the activation of Wnt/β-catenin signalling, further research should develop strategies for their identification. Given the plethora of AhR ligands and its cell type-specific regulatory functions, the role of AhR in the generation of fibrosis could be through different mechanisms. Reporter gene assays could be used to look for other possible pathways that might be involved.

## Data Availability

All experimental data are available upon reasonable request.

## Supplementary Materials

The following supporting information can be downloaded at https://www.mdpi.com/article/10.3390/toxics10110642/s1, Figure S1: Chemokine-cytokine screening, Figure S2: Migration of macrophage cells, Figure S3: Migration of epithelial cells, Figure S4: Effect of conditioned medium, Figure S5: Activation of AhR induced MMP expression in RAW 264.7 cells, Figure S6: Activation of AhR induced MMP expression in RAW 264.7 cells, Figure S7: Activation of AhR induced epithelial EMT marker expression inMLE-12 cells-e-cadherin, Figure S8: Activation of AhR induced epithelial EMT marker expression inMLE-12 cells-fibronectin, Figure S9: Activation of AhR induced epithelial EMT marker expression inMLE-12 cells-Vimentin, Figure S10: Activation of AhR induced epithelial EMT marker expression inMLE-12 cells-β-catenin Figure S11: Activation of AhR induced epithelial EMT marker expression inMLE-12 cells-GAPDH, Figure S12: Activation of AhR target genes by AhR agonists [56,57,58], Table S1: Primer sequences.

## Abbreviations

AhR—Aryl hydrocarbon receptor; EMT—Epithelial—Mesenchymal Transition; MMP—Matrix metalo proteinases; α-SMA—α-Smooth Muscle Actin; ECM—Extra-Cellular matrix; TNF-α—Tumor Necrosis factor-alpha; G-CSF—Granulocyte colony-stimulating factor; MIP-1—Macrophage Inflammatory proteins; TCDD-2,3,7,8—tetrachlorodibenzo-p-dioxin; IP—Indeno pyrene; FICZ—6-formylindolo [3,2-b]carbazole.
