# Supplementary material for: AhR Mediated Activation of Pro-Inflammatory Response of RAW 264.7 Cells Modulate the Epithelial-Mesenchymal Transition
Source: Toxics. 2022 Oct 27;10(11):642. doi: 10.3390/toxics10110642 (PMC9696907; doi:10.3390/toxics10110642)
Supplement: Supplementary file 1 [file toxics-10-00642-s001.zip › toxics-1894983-supplementary.pdf]

Supplementary Data

1.1 TCDD enhances cytokine production in Macrophage RAW 264.7 cells

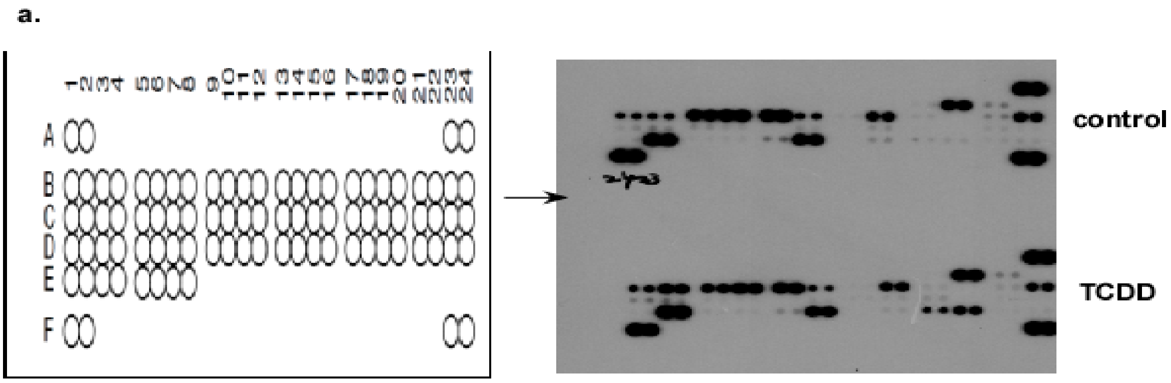

Figure: S1 Chemokine-Cytokine screening. Activation of AhR in macrophage induces inflammatory markers & Fibrosis associated genes. RAW 264.7 cells were seeded (1 X 10^5 Cells/mL) at ~90% confluence on a 60mm plate for 6-8 h before treatment. The cells were treated further for 24h in serum-free DMEM medium containing TCDD (10 $^{-8}$µM). The expression profiles of cytokines/chemokines in RAW 264.7 cells, which was stimulated by TCDD, were analysed and compared with the control cells treated with DMSO.

1.2 AhR agonists induced mouse macrophage cell migration

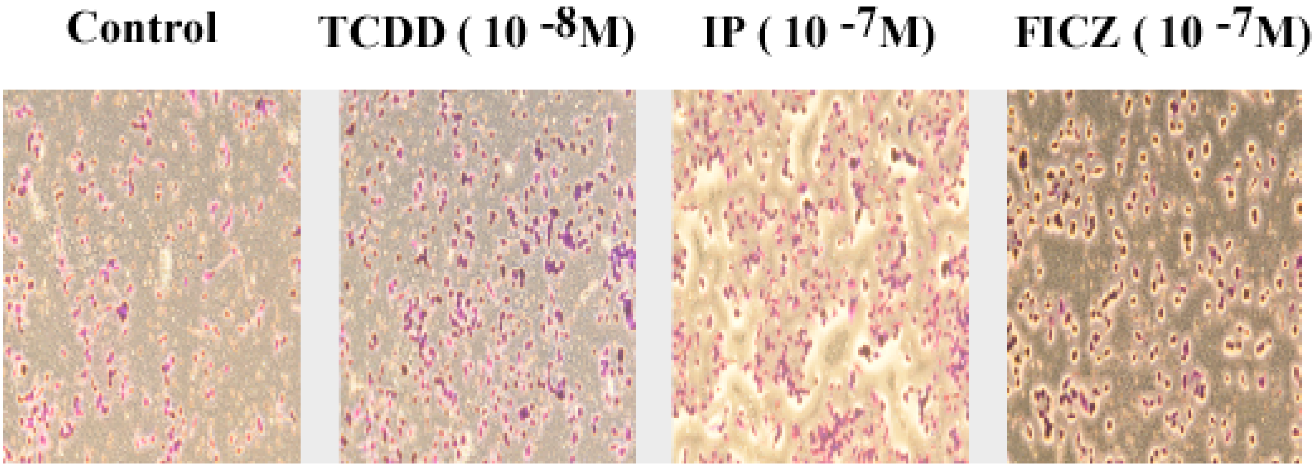

Figure S2. Migration of macrophage cells. In serum-free DMEM media with TCDD (10^{-8} M), IP (10^{-7} M), and FICZ (10^{-7} M), RAW 264.7 cells were cultured for 24 h when images were taken. Crystal violet-stained cells take on a purple hue.

1.3 AhR agonists induced mouse lung epithelial cell migration

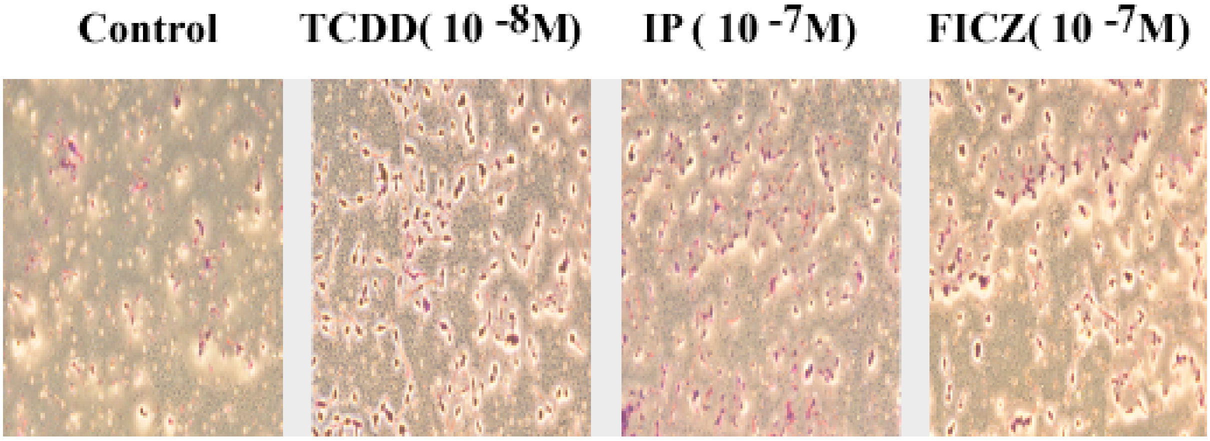

Figure S3. Migration of Epithelial cells. In serum-free DMEM/F12 medium that contained TCDD (10^{-8} M), IP (10^{-7} M), FICZ (10^{-7} M$^{)}$, MLE-12 cells were cultured for 24 h. Images were taken after 24 h. Crystal violet-stained cells took on a purple hue. Quantification of cells were performed as illustrated previously.

1.4 RAW 264.7 cells condition medium induced mouse lung epithelial cell migration

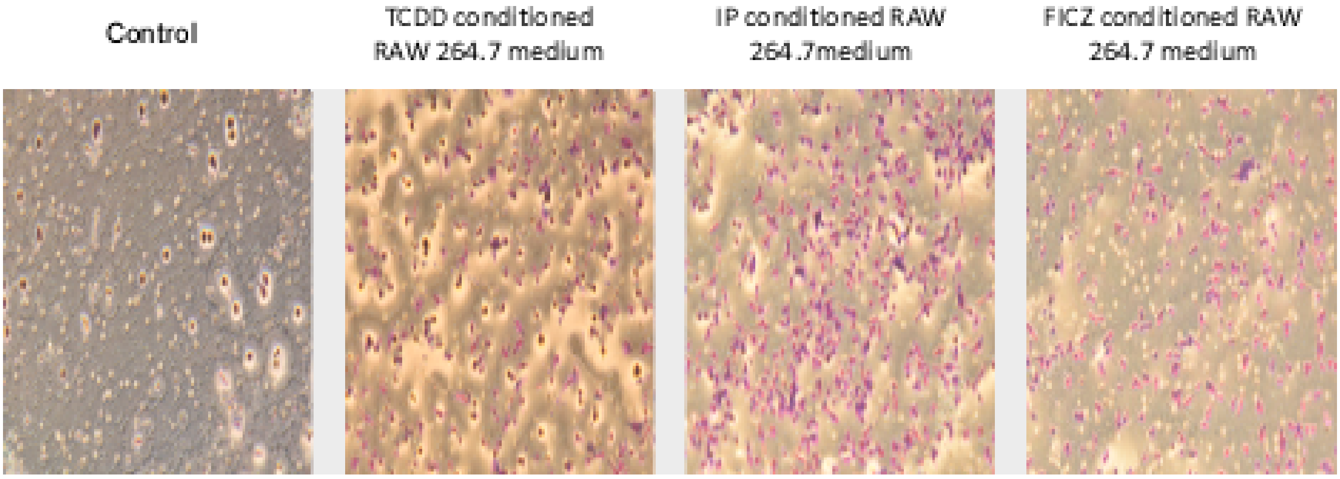

Figure S4. Effect of conditioned medium. a. In serum-free DMEM media with TCDD (10^{-8} M), IP (10^{-7} M), FICZ (10^{-7} M) for 24 h, RAW 264.7 cells were maintained. MLE-12 cells were seeded for 10-12 h before treatment for 24 h with RAW 264.7 conditioned medium. Images were taken after 24 h. Crystal violet-stained cells took on a purple hue.

1.5 Activation of AhR induced MMP expression in RAW264.7 cells

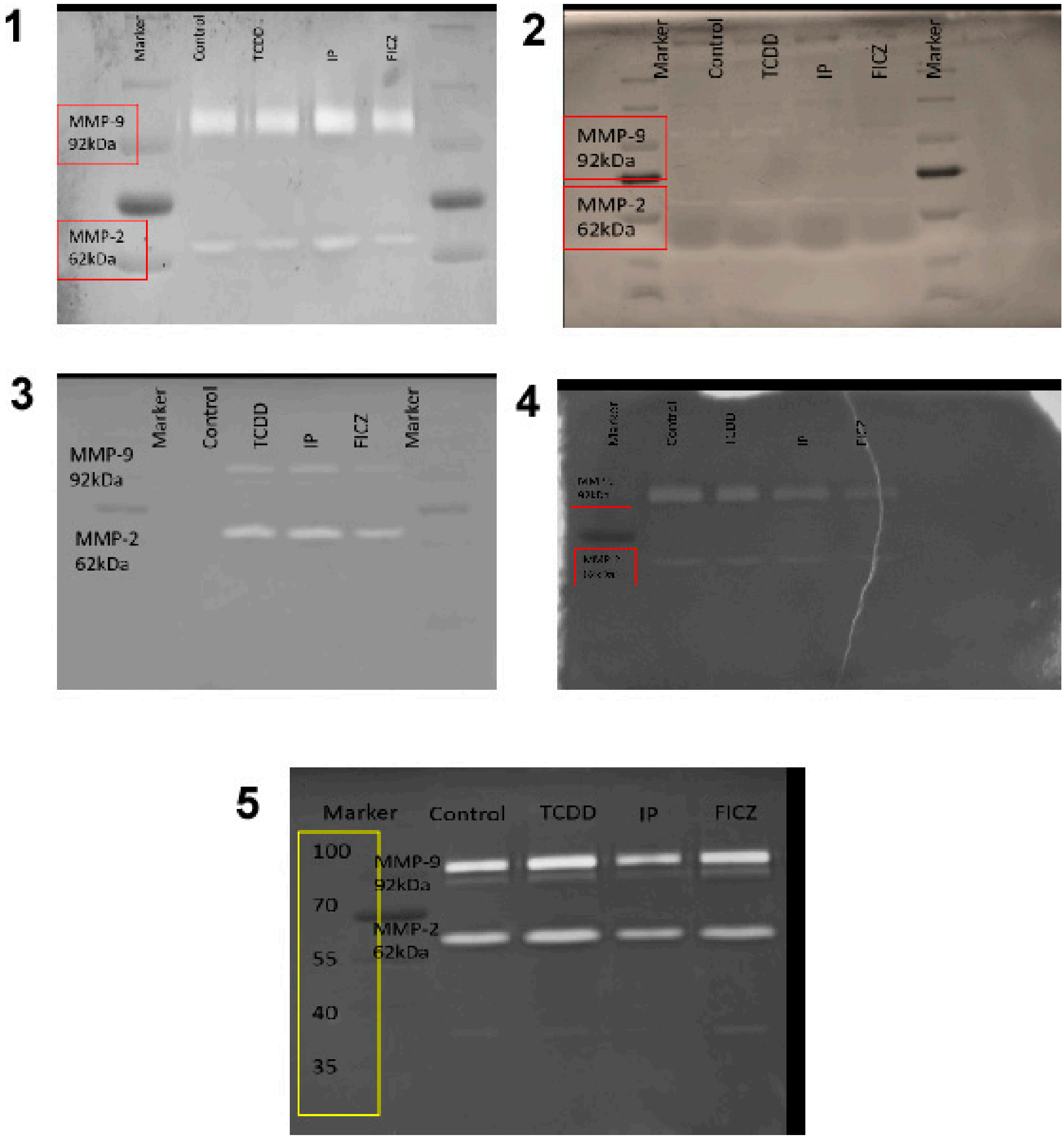

Figure S5. Activation of AhR induced MMP expression in RAW264.7 cells. In serum-free DMEM media with TCDD (10^{-8} M), IP (10^{-7} M), and FICZ (10^{-7} M) for 24 h, RAW 264.7 cells were incubated. The cell supernatant was collected and subjected to SDS-PAGE (containing 0.1% gelatin) electrophoresis.

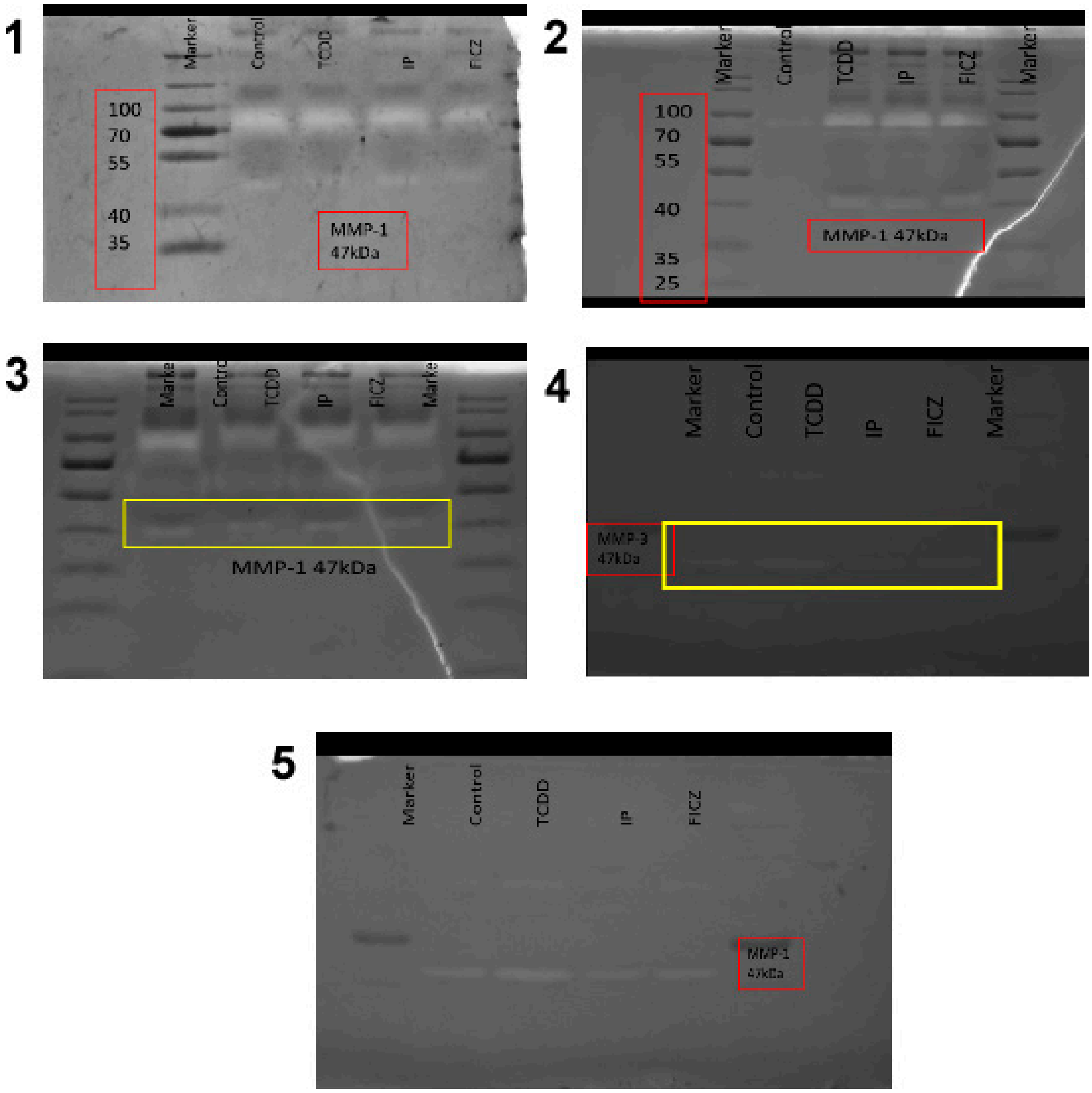

Figure S6. Activation of AhR induced MMP expression in RAW264.7 cells. In serum-free DMEM media with TCDD (10^{-8} M), IP (10^{-7} M), and FICZ (10^{-7} M) for 24 h, RAW 264.7 cells were incubated. The cell supernatant was collected and subjected to SDS-PAGE (containing 1% casein) electrophoresis.

1.6 Activation of AhR induced epithelial EMT marker expression in MLE-12 cells

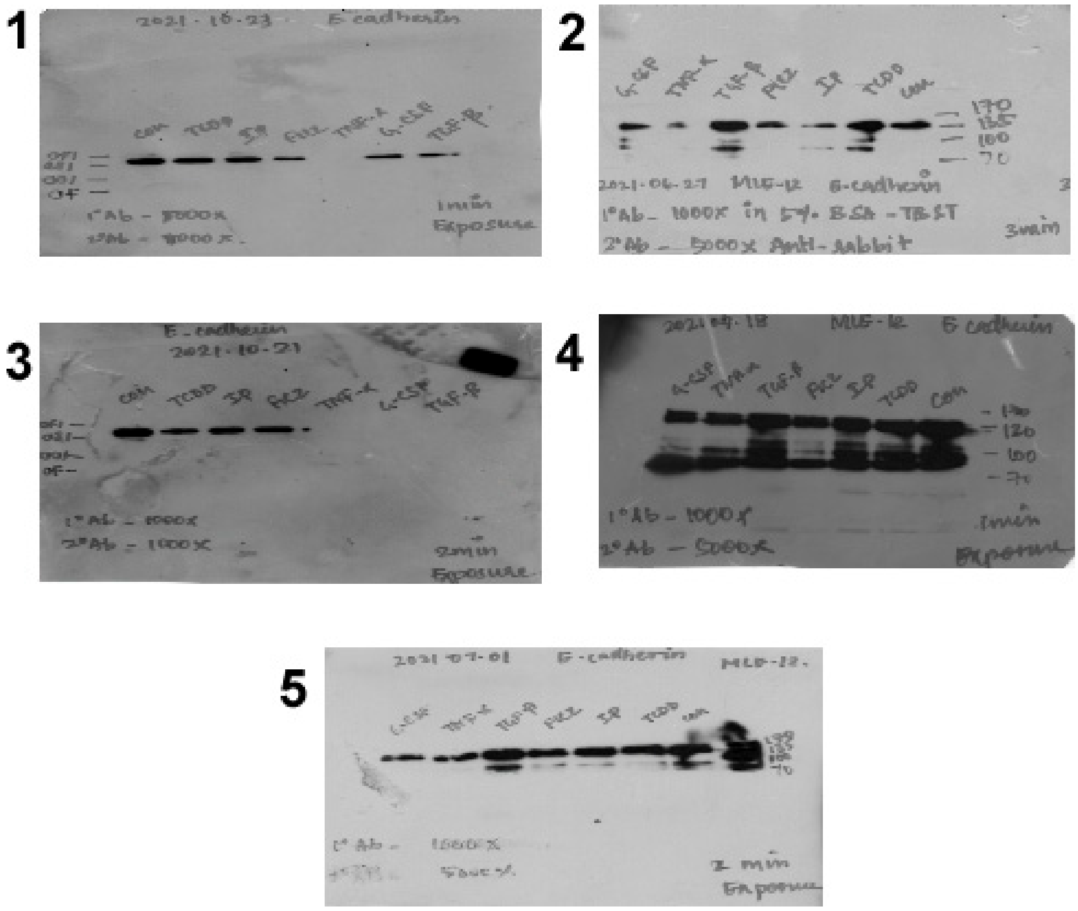

Figure S7: Activation of AhR induced epithelial EMT marker expression in MLE-12 cells- e-cadherin. MLE-12 cells were exposed for 24 h in serum-free DMEM/F12 media with TCDD (10^{-8} M), IP (10^{-7} M), FICZ (10^{-7} M), TNF-α (10 ng/mL), TGF-β (10^{-5} M) and G-CSF (10 ng/mL). The protein was extracted as per protocol and wester blot assay was performed.

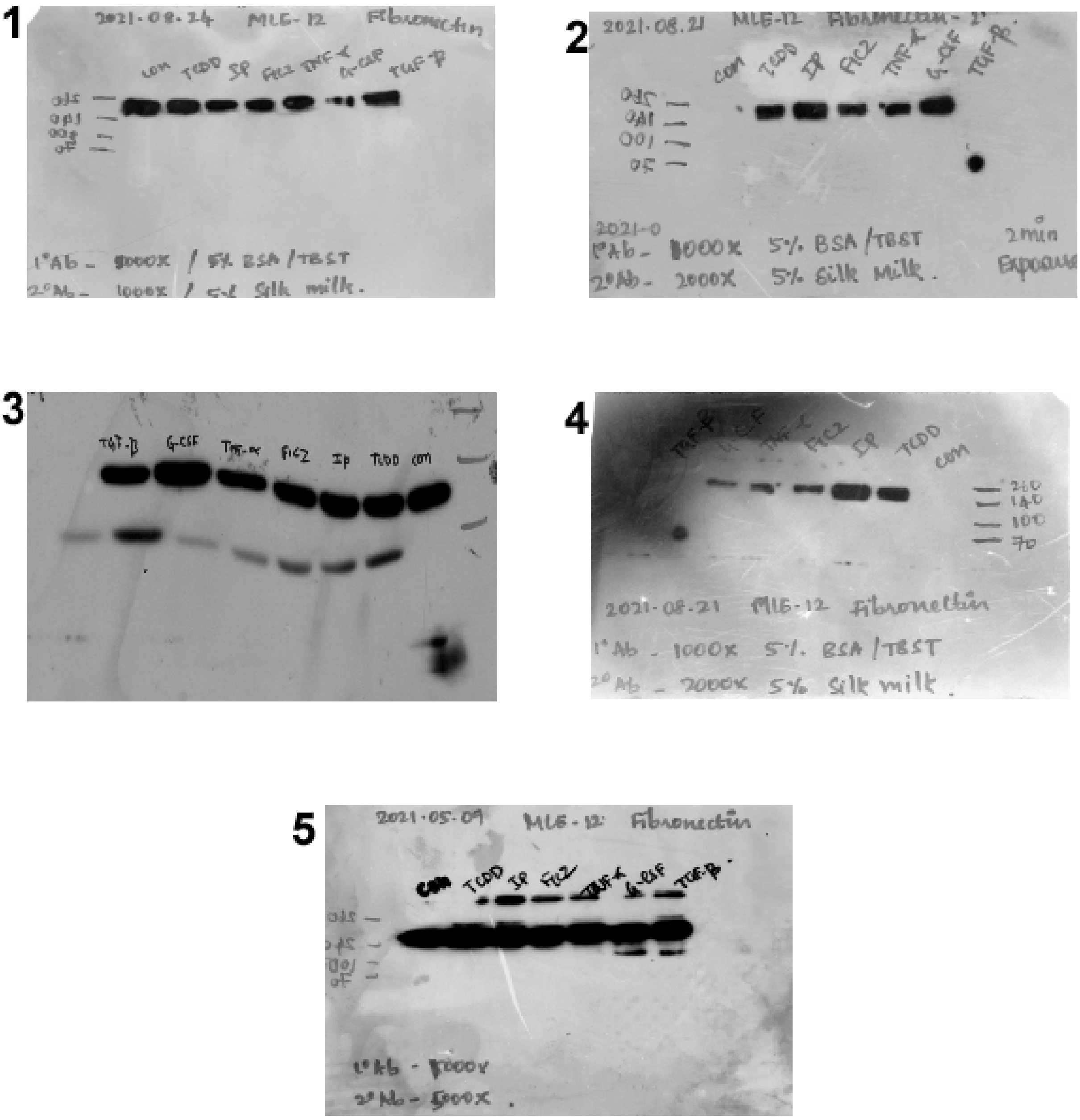

Figure S8: Activation of AhR induced epithelial EMT marker expression in MLE-12 cells- Fibronectin. MLE-12 cells were exposed for 24 h in serum-free DMEM/F12 media with TCDD (10^{-8} M), IP (10^{-7} M), FICZ (10^{-7} M), TNF-α (10 ng/mL), TGF-β (10^{-5} M) and G-CSF (10 ng/mL). The protein was extracted as per protocol and wester blot assay was performed.

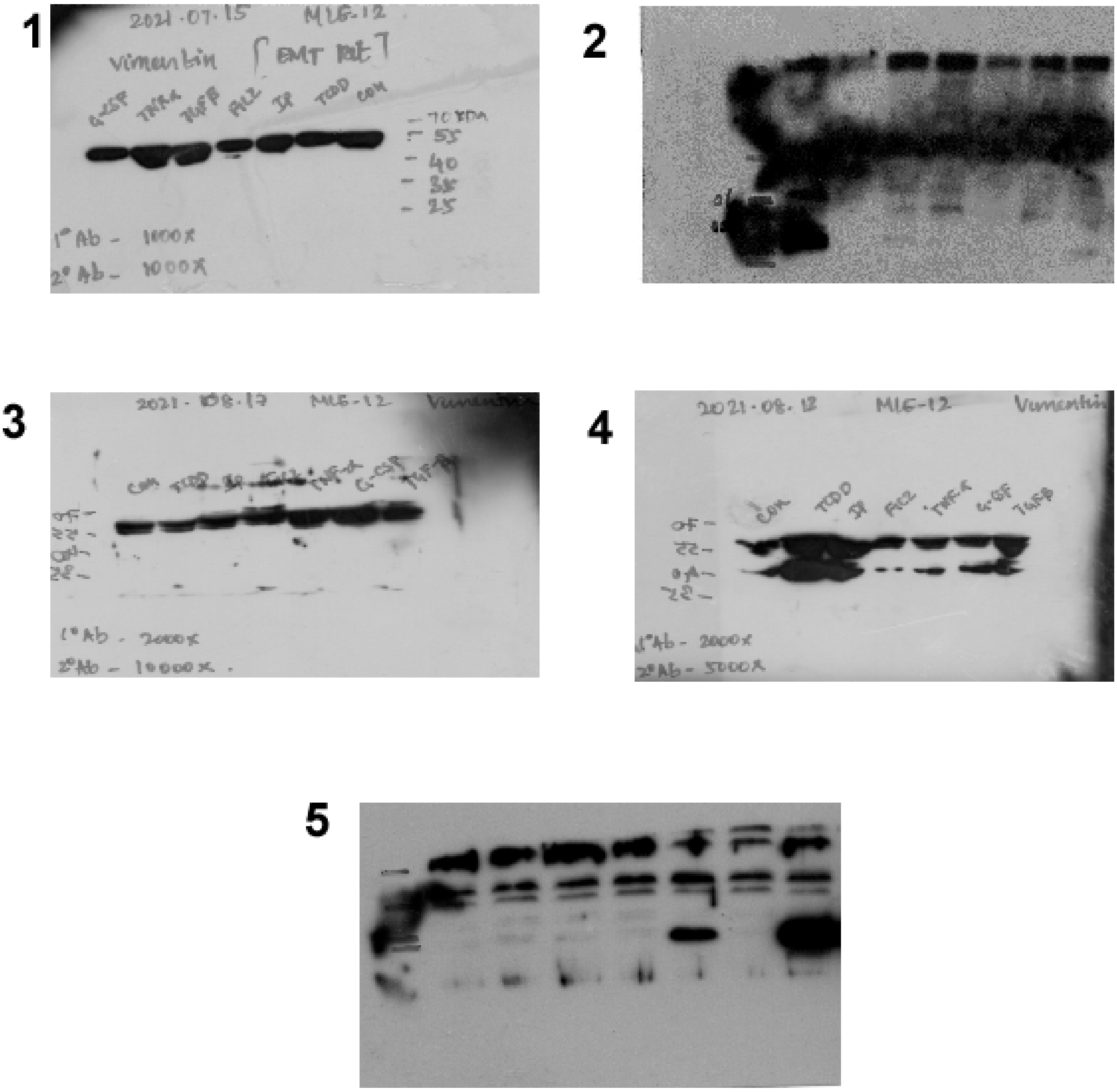

Figure S9: Activation of AhR induced epithelial EMT marker expression in MLE-12 cells- Vimentin. MLE-12 cells were exposed for 24 h in serum-free DMEM/F12 media with TCDD (10^{-8} M), IP (10^{-7} M), FICZ (10^{-7} M), TNF-α (10 ng/mL), TGF-β (10^{-5} M) and G-CSF (10 ng/mL). The protein was extracted as per protocol and wester blot assay was performed.

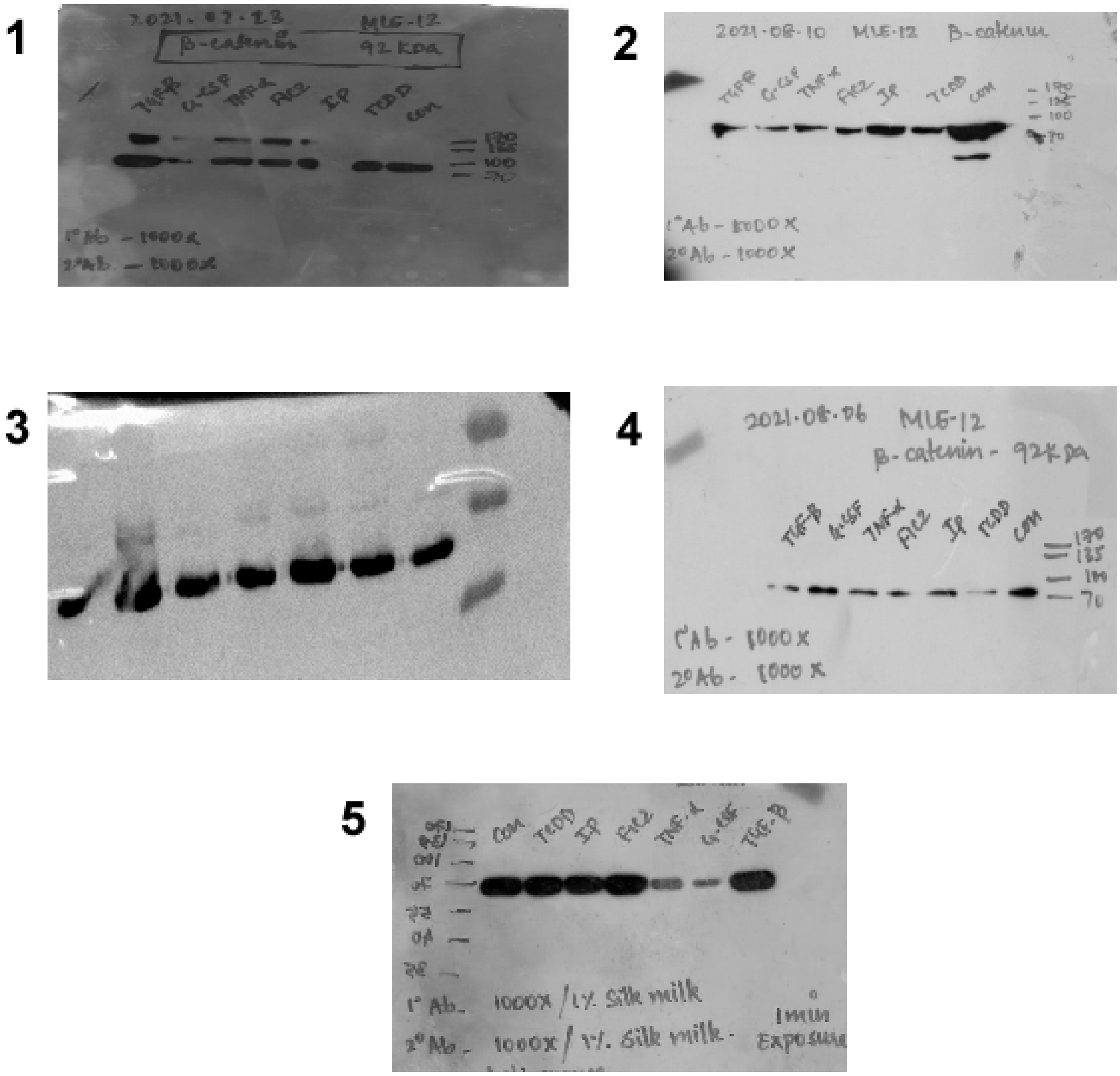

Figure S10: Activation of AhR induced epithelial EMT marker expression in MLE-12 cells-β-catenin. MLE-12 cells were exposed for 24 h in serum-free DMEM/F12 media with TCDD (10^{-8} M), IP (10^{-7} M), FICZ (10^{-7} M), TNF-α (10 ng/mL), TGF-β (10^{-5} M) and G-CSF (10 ng/mL). The protein was extracted as per protocol and wester blot assay was performed.

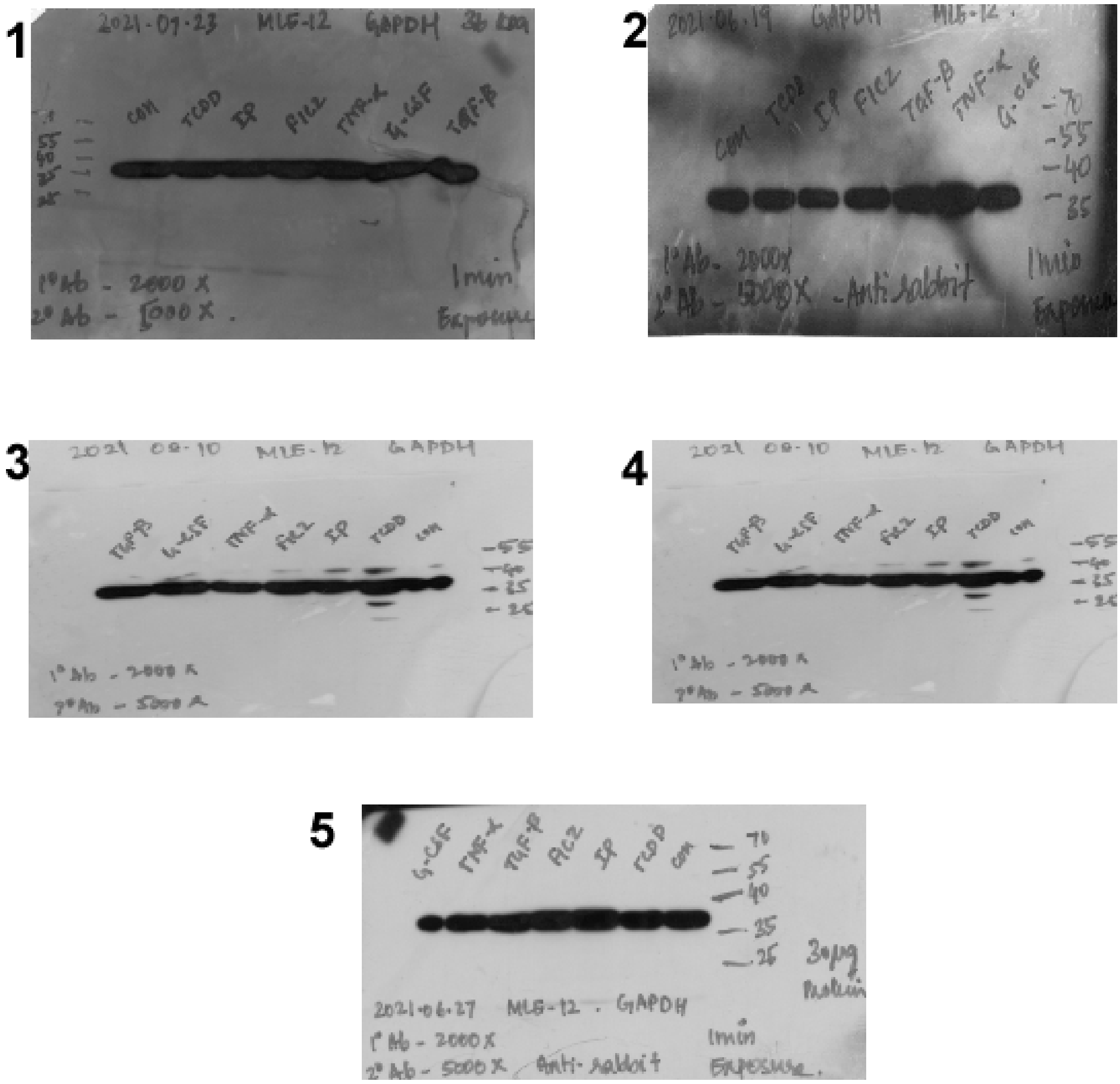

Figure S11: Activation of AhR induced epithelial EMT marker expression in MLE-12 cells- GAPDH. MLE-12 cells were exposed for 24 h in serum-free DMEM/F12 media with TCDD (10^{-8} M), IP (10^{-7} M), FICZ (10^{-7} M), TNF-α (10 ng/mL), TGF-β (10^{-5} M) and G-CSF (10 ng/mL). The protein was extracted as per protocol and wester blot assay was performed.

Activation of AhR target genes by AhR agonists

1.7 RNA isolation and PCR analysis.

To investigate whether the AhR agonists induce AhR target genes- CYP1A1 and CYP1B1, RAW 264.7 cells, 1 X 10^5 cells/mL cells were seeded at ~ 90% confluence on 60 mm plates and cultured for 3 h, 6h and 12 h intervals. After treatment, the cells were collected and RNA was extracted using TRIzol reagent (Invitrogen, 5791 van allen way Carlsbad CA 92008) and reverse transcribed to cDNA using a High capacity cDNA Reverse Transcription kit 4368814 (Thermo Fisher scientific Baltics, Vilnius 02241, Lithuania). PCR was performed using one Taq 2x master mix with standard buffer (M0482S, New England biolabs, Ipswich, MA). The specific gene primers for CYP1A1, CYP1B1and gapdh were shown in Table 1. All the sample data were quantified using Image J software and presented as the mean ratio to gapdh.

Table S1: Primer sequences.

Gene	Forward primer	Reverse primer
Cyp1a1	CCTCATGTACCTGGTAACCA	AAGGATGAATGCCGGAAGGT
Cyp1b1	ACATCCCCAAGAATACGGTC	TAGACAGTTCCTCACCGATG
gapdh	GTATGACTCCACTCACGGCAAAT	GTAGACTCCACGACATACTCAGCAC

Activation of AhR target genes by AhR agonists

Cyp1a1 and Cyp1b1 are two cytochrome P450 enzymes (CYP) that are expressed when the AhR is activated. Many endogenous substances, including cholesterol, steroid hormones, and environmental xenobiotics, are metabolized by Cyp1a1 and Cyp1b1. Both Cyp1a1 and Cyp1b1 are hydroxylases of 17 β-estradiol (E2)(56, 57). Cyp1a1 and Cyp1b1 are known to catalyze the generation of carcinogenic intermediates from a number of polycyclic aromatic hydrocarbons (PAH)(58). To determine whether AhR was activated upon AhR agonist treatment, PCR analysis was carried out for Cyp1a1 and Cyp1b1. As seen in figure S5, both cyp1a1 and cyp1b1 were activated by the agonists.

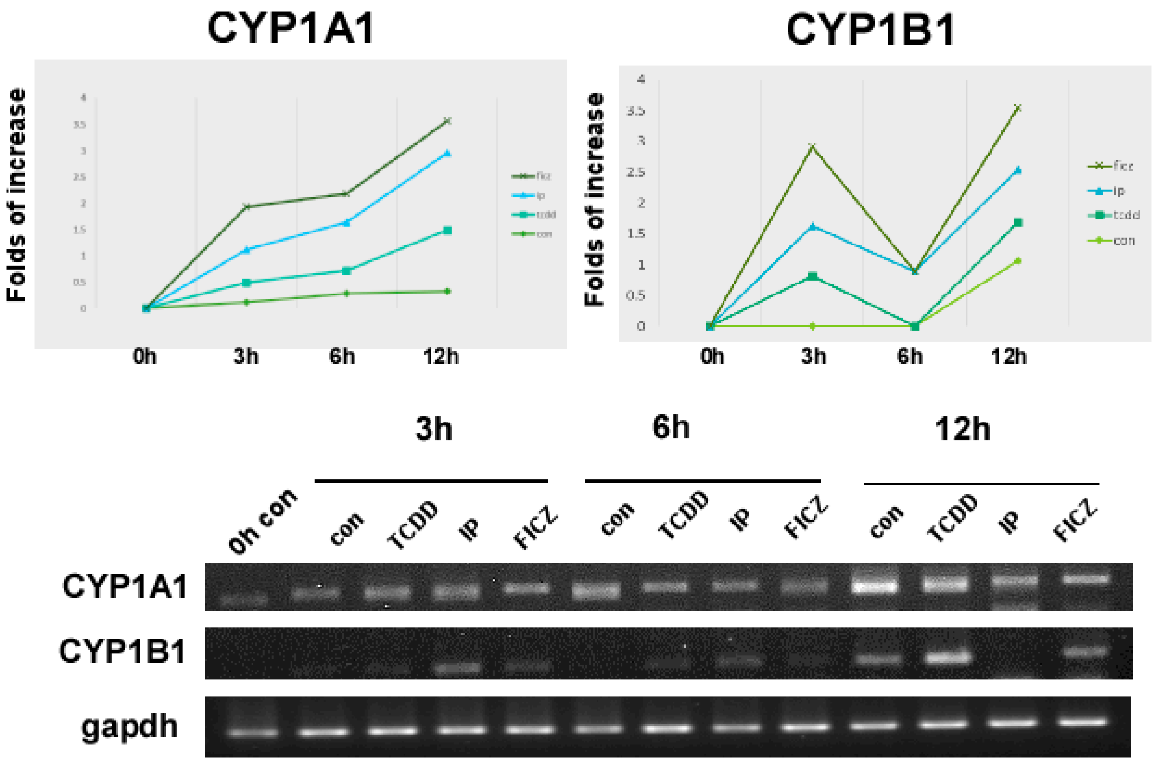

Figure S12. Activation of AhR target genes by AhR agonists. In serum-free DMEM media with TCDD (10^{-8} M), IP (10^{-7} M), and FICZ (10^{-7} M), RAW 264.7 cells were cultured for 3h, 6h, 12h. CYP1A1 and CYP1B1 target genes expression was quantified using image J software.
